# Transient Ischemic Attack Outpatient Clinic: Past Journey and Future Adventure

**DOI:** 10.3390/jcm12134511

**Published:** 2023-07-05

**Authors:** Shima Shahjouei, Homa Seyedmirzaei, Vida Abedi, Ramin Zand

**Affiliations:** 1 Department of Neurology, Milton S. Hershey Medical Center, Penn State Health, Hershey, PA 17033, USA; 2 Department of Neurology, Neurosurgery, and Translational Medicine, Barrow Neurological Institute, St. Joseph Hospital, Phoenix, AZ 85013, USA; 3 School of Medicine, Children’s Medical Center Hospital, Tehran University of Medical Sciences, Dr. Qarib St., Tehran 14155-34793, Iran; homa_sdmr@yahoo.com; 4 Interdisciplinary Neuroscience Research Program (INRP), Tehran University of Medical Sciences, Keshavarz Blvd., Tehran 14166-34793, Iran; 5 Department of Public Health Sciences, College of Medicine, The Pennsylvania State University, Hershey, PA 17033, USA; vidaabedi@gmail.com; 6 Department of Neurology, College of Medicine, The Pennsylvania State University, Hershey, PA 17033, USA

**Keywords:** ischemic attack, transient, ischemic stroke, brain ischemia, ambulatory care facilities, TIA clinic, system dynamics

## Abstract

A transient ischemic attack (TIA), a constellation of temporary neurological symptoms, precedes stroke in one-fifth of patients. Thus far, many clinical models have been introduced to optimize the quality, time to treatment, and cost of acute TIA care, either in an inpatient or outpatient setting. In this article, we aim to review the characteristics and outcomes of outpatient TIA clinics across the globe. In addition, we discussed the main challenges for outpatient management of TIA, including triage and diagnosis, and the system dynamics of the clinics. We further reviewed the potential developments in TIA care, such as telemedicine, predictive analytics, personalized medicine, and advanced imaging.

## 1. Introduction

**Figure 1:**
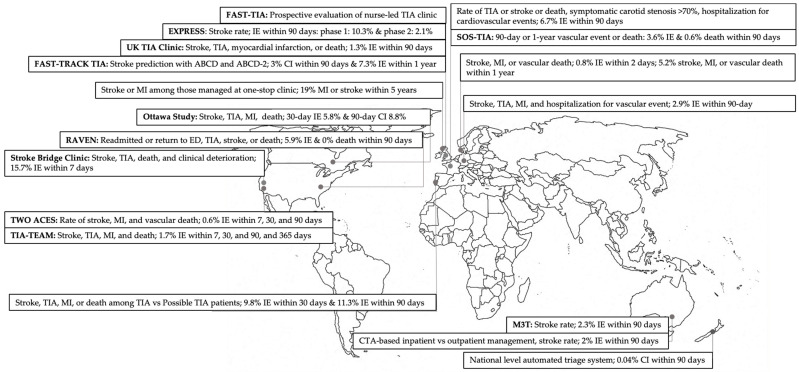
Summary of studies presenting outpatient TIA care across the world. TIA, transient ischemic attack; MI, myocardial infarction; IE, ischemic event (TIA or stroke); ED, emergency department; CTA, computed tomography angiogram.

A transient ischemic attack (TIA) is manifested by transient focal neurologic dysfunction due to loss of blood flow to the brain, spinal cord, or retina without evident acute infarction or tissue injury [1]. Per definition, TIA symptoms resolve within 24 h, or even much shorter, making their diagnosis and management challenging. However, TIA precedes about 20% of stroke events [2], and appropriate strategies should be obtained to prevent detrimental complications in patients with TIA. Two decades ago, the EXisting PREventive Strategies for Stroke (EXPRESS) study [3] proposed referral to outpatient clinics for acute management of patients with cerebral ischemia. Subsequently, various TIA outpatient care settings were introduced around the world, offering alternative modalities of care either through or independent of emergency services [4,5,6,7,8]. While the concept of a TIA clinic has gained acceptance in recent years as a valuable tool for managing patients with TIAs, there may still be some debate surrounding its optimal structure, implementation, safety, and effectiveness. Currently, in the United States, many patients with a TIA or a minor ischemic stroke (mIS) are hospitalized to receive a full workup. The purpose of this narrative is to overview the present models of rapid access TIA clinics, their challenges, and the upcoming paradigm shift in defining and providing care for patients with TIA and minor stroke (Figure 1). This review was inspired by the studies retrieved in our previous systematic review [9], but we extended our search to include more recent models of outpatient TIA care.

## 2. Models of Care for TIA Patients

Although published years later, the FAST-TRACK TIA Clinic (1992–2004) [10] is among the earliest reports on outpatient management of patients with TIA in Glasgow, United Kingdom. Experienced consultant stroke physicians referred patients to the clinics twice per week, with a median 15-day delay from the onset of symptoms. This referral system reported a 7.3% risk of recurrent stroke within one year (Table 1). The EXisting PREventive Strategies for Stroke (EXPRESS) study [3] reported the outcome of TIA patients managed in an outpatient setting out of a population-based dataset (the Oxford Vascular Study, OXVASC, in the United Kingdom). During the first phase (2002–2004), patients with TIA and mIS were referred to a weekday-only hospital outpatient clinic by fax. In the second phase (2004–2007), daily TIA clinics no longer required making appointments, and they guaranteed the immediate start of preventive medications in the clinic. This strategy reduced the median delay of visiting patients in the clinic to less than a day (versus three days in phase 1) and the median delay to the first prescription to one day (versus 20 days in phase 1). As a result, an 80% reduction of recurrent cerebral ischemia from 10.3% in phase 1 to 2.1% in the second phase was observed. The ten-year follow-up of the EXPRESS study [11] supported lower overall stroke risk and the risk of disabling/fatal strokes, as well as a higher disability-free life expectancy for patients who participated in the second phase. In another British outpatient model in London (2003–2006) [12], referrals from local primary care and in-hospital sources were accepted through telephone or a dedicated fax number. Patients with suspected symptoms of anterior circulation were triaged by a specialist nurse through the FAST-TIA protocol [12] during business hours. The median time from referral to assessment was 3 days (event to assessment median of 7 days), and about a third of the referred patients were seen on the first day.

By setting 15 exclusion criteria, including multiple items, such as age under 40, presence of vertigo, balance difficulty without weakness, or any arrhythmia, the authors reported an 86% rate of cerebral ischemia among referred patients, significantly above the national average of 55%. A study of a larger cohort of patients referred to the daily TIA clinic of a United Kingdom general hospital (2010–2012) [22] was consistent with national average rates and reported a final diagnosis of cerebral ischemia only among half of the cohort. The overall 90-day stroke risk of 1.3% among this subset of patients from the UK TIA clinic was comparable to EXPRESS.

In Lothian, Scotland [23], patients with suspected neurology symptoms were referred either by letter or fax (2005–2007) or telephone or email (2007–2013) to a one-stop clinic. All patients underwent same-day brain imaging and cardiac workups. Among those patients with the diagnosis of cerebral ischemia (60% of the cohort), 19% had a stroke or myocardial infarction over 5 years, compared to 10% among other patients.

In France, the SOS-TIA (2003–2008) [5,16] provided a 24-hour toll-free telephone consult and rapid access to a hospital clinic. Thousands of family physicians, cardiologists, neurologists, and ophthalmologists in Paris and surrounding regions were informed and instructed to refer patients with cerebral ischemia to this TIA clinic. About half of these patients were seen in the clinic within the first day of symptom onset, and all patients received emergency assessments within 4 h of admission. About three-fourths of the patients were discharged home on the same day, and the 90-day stroke risk was 1.24%, much less than the prediction of the ABCD2 score (5.96%). Under the influence of the SOS-TIA model, another study (2012–2013) [24] dedicated two monitored beds inside the stroke unit. With support from the ED system, this model aimed to offer comprehensive imaging and other evaluations within 12 h of the onset of symptoms. The median delay to arrival at the TIA clinic in this study was 8 h, and referral by office-based physicians was the main factor resulting in a delay of over 12 h for the clinic evaluation after a TIA incidence. In another French model [18], patients with symptoms of TIA were primarily examined by an emergency physician. Patients with total recovery from the symptoms and routine investigations were managed as outpatients under the supervision of vascular neurologists within the next 8 to 15 days. Within 90 days of the index event, 1.7% of the patients managed in the outpatient setting presented with a stroke, and 5% had a subsequent TIA.

In Australia, the Monash Transient Ischemic Attack Triaging Treatment (M3T) protocol (2004–2007) [4] offered an emergency department (ED) physician rapid evaluation and management of patients with TIA. Patients were referred to urgent outpatient clinics through stratification by vascular mechanism without dependence on the ABCD2 score. Using a carotid ultrasound study on over 85% of patients within the first two days, the M3T model resulted in a 1.5% risk of stroke within 90 days. Another study from Australia (2012–2016) [13] considered urgent computed tomography angiography (CTA) in addition to semi-urgent (within 3–4 days) magnetic resonance imaging (MRI) screening to manage TIA. The risk of stroke among the outpatient cohort was 2% during the 90 days of follow-up. However, only about half of the admitted patients met the predefined criteria. Moreover, a considerable number of the clinic patients (22%) were either not referred (mainly due to human error) or referred but not seen at the clinic (11%). In Sydney, the Royal North Shore Hospital [25] defined a one-stop, twice-weekly multidisciplinary clinic to avoid the hospitalization of patients with cerebral ischemia. Patients received primary imaging and management in the ED. During the first year, only patients with ABCD2 < 4 were eligible to receive care in the clinic, while after that, all patients were referred to this service. The average ED-to-clinic presentation was 3.9 days, with an overall 2146.5 bed days and an AUD$1,180,575 saving over a period of 24 months.

In the United States, the TWO ACES study (TIA Work-up as Outpatient Assessment of Clinical Evaluation and Safety, Sandford, 2007–2009) [8] proposed the disposition of patients with TIA from ED based on their ABCD2 score and severity of carotid stenosis. According to this protocol, 157 (70%) patients were discharged to the TIA clinic, with a median delay of 4 days from symptom onset. The subsequent stroke rate of TIA clinic patients at 7, 30, and 90 days was 0.6%. In a more recent protocol from the same center (TIA-TEAM, TIA triage in the emergency department using acute MRI) [19], all patients received a consultation by a neurology resident. Decision-making on patients’ dispositions was based on acute neuroimaging. With a median interval of 16 h between symptom onset and MRI, over 60% of the patients were discharged from the ED. The recurrent stroke rates at 7 and 90 days were 1.1%. Under the influence of the TWO ACES study and in collaboration with Stanford University, in 2013, Virginia Mason Franciscan Health [26] utilized an ED triage tool to refer low-risk patients from ED (2016) or ED and primary care (2017–2018) to a rapid outpatient access TIA clinic within 2–3 days. After completing brain imaging, patients were scheduled for 90-minute appointments with a nurse practitioner and a neurologist. With a mean of 3 patients per month (a total of 99 patients over 3 years), this study reported a final diagnosis of TIA or stroke in 51% of patients and two stroke-related admissions within 90 days. In the stroke bridge clinic model (2013–2014) [17], patients with a TIA or mIS with a national institutes of health stroke scale (NIHSS) ≤ 3 within 6 h of symptoms’ onset were prospectively randomized by a vascular neurologist to be admitted or receive outpatient care within 72 h of discharge from ED. This study excluded patients with anterior circulation ischemia and significant extracranial carotid stenosis. The outcome of patients in the two arms was not significantly different regarding TIA recurrence, subsequent stroke, or death. RAVEN (Rapid Access Vascular Evaluation—Neurology, 2016–2018) [7] proposed an algorithm for stratifying the patients who presented to the ED with TIA or mIS (NIHSS ≤ 5) to assess the feasibility and safety of outpatient management within 24 h of ED discharge. Of note, vascular imaging was not among the primary referral workups in the RAVEN criteria. Among the patients with confirmed cerebral ischemia (34% of those referred to the TIA clinic), 19% returned to the ED within 90 days, 5% had a recurrent TIA, and 1% had a subsequent stroke. Compared to admitted patients, patients managed at RAVEN Clinic averted a total cost of $764,000 and 208 hospital bed days in the accounting year 2017 [7,27].

The Aarhus TIA study in Denmark (2007–2008) [21] defined a referral system to the acute TIA team directly from the primary care provider or emergency medical services (EMS), bypassing the ED. Patients with crescendo TIA or TIA within the last 48 h of medical attention were admitted to the stroke unit, while the rest (35% of patients) were referred to outpatient clinics. By visiting 72% of these patients within the first week, this study reported a recurrent stroke rate of 1.6% within 7 days and 2% within 90 days after the index event. The same team [28] compared the outcome of patients with TIA who were referred to the Aarhus TIA clinic between 2013 and 2014 (N = 1076, 36% with a final diagnosis of cerebral ischemia) to contemporary hospitalized controls, matched for age, sex, stroke severity, multiple variables related to the treatment with thrombolysis, and subtype of diagnosis. The authors reported a shorter acute length of stay, a lower mortality rate within 365 days, and higher process performance measures (antiplatelet therapy within 2 days, anticoagulation therapy within 14 days, brain imaging on the same day, and imaging of the carotids within 4 days) among patients who were managed in an outpatient setting.

In Canada, one study [20] used the ABCD2-based ED triaging tool to stratify patients with TIA for outpatient management. Although all strata received the same care, the interval to be seen in the outpatient was determined by this risk scoring, from more than 14 days of the index TIA in patients with ABCD2 < 4 to less than a week in those with ABCD2 ≥ 6. The risk of a subsequent stroke within 90 days in all strata was 3.2%.

In Germany, Klinikum Harlaching stroke center [29] introduced an outpatient TIA clinic for the management of low-risk TIA or mIS patients during business hours. Compared to the 5.7% overall 90-day stroke risk predicted by the ABCD2 score, this approach resulted in a stroke rate of 2.9% among the subgroup of patients with a definite TIA or minor stroke. In Portugal, an outpatient TIA clinic [14] serving once a week reported a stroke recurrence rate of 3.1% at 30 days and 4% at 90 days. Except for primary investigations offered in the ED, the evaluation and management of the patients were completed at the TIA clinic.

In New Zealand, the Australian National Stroke Foundation introduced a triaging system consisting of ABCD2 and a set of high-risk indicators [30]. This national system is supported by an electronic decision support tool [31] to guide general physicians on the diagnosis and treatment of TIA and stroke. Patients with ongoing neurological symptoms, ABCD2 > 3, recurrent events in the last 7 days, the presence of atrial fibrillation, and the use of anticoagulants were considered high-risk and were seen within the first 24 h by a specialist at hospitals. Low-risk ones were started on antiplatelet therapy and managed in TIA clinics or the community. This system resulted in a similar risk of subsequent stroke but reduced recurrent TIA or stroke risk within 90 days compared to usual care without an electronic support system. Details of the outpatient TIA care models are available in Table 2.

## 3. Challenges of Outpatient TIA Management and Possible Solutions

### 3.1. Definition, Triage, and Diagnosis

The primary definition of TIA emphasized the temporary nature of the symptoms to distinguish it from a minor stroke. Accordingly, the time-based definition of TIA refers to a constellation of symptoms that persist for less than 24 h, making its diagnosis challenging for patients and healthcare providers. Deploying imaging in the assessment of patients with suggestive symptoms of TIA revealed the presence of cerebral ischemia in up to half of the patients [32,33], even in the early hours, or unremarkable imaging in some patients with more prolonged neurological symptoms [32,34,35]. The result of our previous study [36], similar to other studies [7,22,31], indicates that about one-third to half of patients with suggestive symptoms have a final diagnosis of cerebral ischemia. Despite many attempts to introduce various diagnostic tools, the diagnosis of TIA remained at the center of attention. Multiple clinical scoring systems, such as ABCD2, imaging modalities, or a combination of different strategies [31,37], have been suggested to increase the efficacy of TIA diagnosis versus its mimics. Still, none were satisfactory on a larger scale.

There has been some debate in recent years about whether the term “TIA” should be retired or redefined, as some research suggests that the risk of subsequent stroke may be higher than previously thought or that the labeling is associated with a high misdiagnosis rate. While there are arguments for and against retiring the concept of TIA, it is generally agreed that the diagnosis and management of patients with TIA-like symptoms should be based on a comprehensive assessment of their individual risk factors, symptoms, and imaging findings. Ultimately, the decision to retire or redefine the concept of TIA should be based on a careful evaluation of the available evidence and the potential implications for patient care. While the concept of TIA may evolve over time, the need for prompt and accurate diagnosis and management of patients with TIA-like symptoms remains a critical component of neurologic care.

### 3.2. System Dynamics of Outpatient TIA Clinics

The system dynamics of outpatient TIA clinics, also referred to as the “learning health systems” strategy, can refer to the various factors and interrelationships that affect the functioning and performance of these facilities, resources, and metrics. Some key factors that influence the dynamics of outpatient TIA clinics can include: (a) demographics and patient demand; (b) staffing and scheduling; (c) availability of medical imaging (CT, MRI, and echocardiogram); (d) funding and reimbursement; and (e) regulations and policies.

As mentioned above, studies attempted to deploy strategies to maximize the efficiency of referral systems and minimize waiting periods and loss of follow-up. However, our prior meta-analysis [9] demonstrated a no-show rate of 36% among referred patients with suspected TIA or minor stroke but a minimal risk of complications while waiting for an outpatient visit. Choice of appropriate brain imaging and cardiac workups, administration of first-line medications, such as aspirin, deciding whether and when a neurologist should be involved in diagnosis, and the potential roles of nurse practitioners are other challenges in defining the optimal referral system for outpatient management of patients with TIA [31,38,39].

Similar to the outcomes in patients with stroke, growing evidence suggests a decreased risk of recurrent ischemic events among patients with TIA who received antiplatelets. The addition of ticagrelor (THALES trial) [40] or clopidogrel (FASTER, CHANCE, and POINT trials) [41,42,43] to aspirin on day one, compared to those receiving aspirin alone, has been reported to have a lower risk of subsequent ischemic stroke but a higher rate of hemorrhagic events. Among the outpatient TIA clinic models, the majority of recent studies reported prescription of antiplatelets in the ED and prior to discharge to the TIA clinic (Table 2) [4,7,13,17], but the protocol of care varies among centers. In some models, medication was prescribed after a full assessment [15], and this information is missing from earlier reports [10].

Diffusion-weighted MRI is the preferred method for the detection of cerebral ischemia in the acute phase [44]. However, in most centers, patients received an initial CT scan in the ED, and MRI or more complex vascular imaging were offered later for selected high-risk patients. As imaging became an entangled component of TIA diagnosis and prediction of the outcome, centers attempted to optimize the timing and protocol of imaging. In some models, multimodal MRI was offered in the ED [19], while in other models, carotid duplex and transcranial Doppler were offered first, and only a few patients received CTA or MRA [7]. The majority of the models defined an algorithm for referral to the outpatient clinic and completion of the imaging within a certain period with respect to the risk for recurrent ischemic events (Table 2).

Rapid-access outpatient clinics are promising models to mitigate the length of hospital stays and total cost to the health system [4,45,46,47]. However, the cost of maintaining the clinic with trained staff and on-site facilities should be considered in centers with fewer patient referrals.

### 3.3. Outcome Measures and Risk of Life-Threatening Complications

Although earlier models of TIA clinics were concerning for a high rate of stroke and other major complications after the index TIA [3], our recent study offered a comparable risk of subsequent stroke among patients with minor cerebral ischemia who were treated in an outpatient setting compared to hospitalized patients [9].

## 4. Tele-Stroke Centers

Like other areas of medicine, telemedicine intends to extend access and optimize the quality of care for patients with cerebral ischemia. The COVID-19 pandemic was a turning point for many healthcare systems to implement telemedicine in a regular flow of patient care [48]. At the beginning of the pandemic, public anxiety about COVID-19 and global policies toward postponing non-urgent care discouraged patients with mild cerebrovascular symptoms from seeking care [49].

A study of 18 centers with rapid TIA pathways across the globe demonstrated that the majority (63%) of these clinics offered telehealth exclusively during the pandemic [50]. Outcome measures were not inferior to in-person care models [51,52]. Despite this, limitations toward physical examination, technical difficulties, security breaches, and regulatory issues should be addressed before transformation to tele-stroke exclusive models [53].

## 5. Ongoing Clinical Trials for Outpatient TIA Management

Evaluation of the Effectiveness of a City Hospital Care Network for the Care of Patients with Transient Ischemic Accident (AIT-AMBU-GRE; NCT05216198) [54] aimed to evaluate the feasibility of outpatient management for patients with TIA who were referred from the emergency room. Investigators considered the percentage of patients who have completed all required examinations as their outcome measure. The Feasibility Study on the Medical and Economic Consequences of Outpatient Management of TIAs and Minor Strokes (MEDECO-AIT; NCT03605355) [55] will collect economic outcomes (i.e., direct and indirect costs) alongside clinical outcomes (i.e., cerebrovascular events) of patients who were managed in the TIA clinic in Toulouse Hospital, France, in a prospective manner. The average cost-effectiveness ratio at 3 months will be the primary outcome of this trial. Telestroke for Comprehensive Transient Ischemic Attack Care in Acute Stroke Ready Hospitals (TELECAST-TIA; NCT03724110) [56] will compare diagnostic stroke evaluation, secondary stroke prevention, health screening and evaluation, stroke education, inpatient complications, and stroke recurrence rates pre- and post-initiation of a specialist tele-stroke inpatient rounding service. In addition, patient and provider satisfaction scores, transfer patterns, and a cost analysis will also be reported by TELECAST-TIA.

## 6. Future Direction

TIA care will likely undergo several changes in the coming years due to technological advancements and healthcare delivery models. Some potential developments include: (A) Telemedicine: As discussed above, with the rise of telemedicine, TIA patients may be able to receive care remotely, which can help improve access to care, reduce costs, and increase convenience. (B) Predictive analytics and continuous self-monitoring technologies: Predictive analytics can help identify patients at risk of stroke, allowing for early intervention and prevention as well as participatory medicine, where patients are empowered to reduce and help manage their risk factors. (C) Personalized medicine: Advances in genetic testing and precision medicine may enable more personalized treatment plans for TIA and stroke patients based on their individual risk factors and genetic profiles. (D) Improved imaging technology and finding new biomarkers: Advances in imaging technology, such as CT scans and MRIs, and other laboratory advancements can help improve the accuracy of TIA and stroke diagnosis and enable more targeted treatment. (E) Multidisciplinary care teams: Collaborative care models involving neurologists, cardiologists, and other specialists may become more prevalent, allowing for more comprehensive and coordinated care for TIA patients. (F) Integration with larger healthcare systems: As healthcare consolidation continues, especially in the US, TIA clinics may increasingly become part of larger healthcare networks, such as hospitals or accountable care organizations. This could provide TIA clinics with access to greater resources and expertise, as well as a larger patient population to serve. Overall, the future of TIA care is likely to involve a greater focus on prevention, early intervention, personalized and participatory care enabled by technology and innovative healthcare delivery models, and patient empowerment.

## 7. Conclusions

Despite some challenges, well-established TIA clinics are safe and effective in providing prompt evaluation and treatment. The future of TIA clinics, especially in the United States, is likely to be shaped by a combination of changes in healthcare policies, technology, integration with larger healthcare systems, and patient needs. By focusing on prevention, leveraging telemedicine, and embracing new medical technologies, TIA clinics can continue to provide high-quality care to patients with TIA and mild stroke.

## Figures and Tables

**Table 1:** Outcome of the outpatient TIA care models.

Study, Publication Year; Country	Recruitment	Study Design	Included Patients	Definition of mIS	Definition of TIA	Sample Size (N)	2-Days Stroke Risk (%)	7-Days Stroke Risk (%)	30-Days Stroke Risk (%)	90-Days Stroke Risk (%)	Mean Age (Year), Men (%)	Hypertension (%)	Diabetes (%)	Myocardial Infarction (%)	Ischemic Heart Disease (%)	Dyslipidemia (%)	Atrial Fibrillation (%)	Carotid Stenosis (%)	Smoking (%)	Prior TIA (%)	Prior Stroke (%)	ABCD2
Chang et al. (RAVEN), [7] 2019; USA	2016–2018	R	TIA andmIS	-	Clsc	101	-	-	-	0.9	-, -	-	-	-	-	-	-	-	-	-	-	-
Cheong et al., [13] 2019; Australia	2012–2016	P	TIA	NA	T. B.	306	0.0	0.3	0.3	1.3	68, -	58.0	18.0	-	12.0	48.0	10.0	-	17.0	-	-	Median: 4 [3–5]
Correia et al., [14] 2015; Portugal	2004–2009	P	TIA	NA	T. B.	258	-	-	3.1	4.0	68, 59.7	73.5	17.1	-	9.7	47.3	8.9	76.7	67.7	8.5	2.3	>4:72%
Dutta et al., [15] 2016; UK	2010–2012	R	TIA and mIS	<24 h, Pos Imaging	Clsc	529	-	-	-	1.3	-, -	-	-	-	-	-	-	-	-	-	-	>4:31.5%
Harrison et al. (Fast-Track TIA), [10] 2010; UK	1992–2004	R	TIA	NA	Clsc	795	-	-	-	3.0	76, 43.0	29.2	8.2	-	-	-	-	-	-	-	-	-
Lavallée et al. (SOS-TIA), [16] 2017; France	2003–2008	P	TIA and mIS	>24 h	T. B.	1850	-	-	-	3.6	63, 52.0	65.1	10.7	9.3	9.3	39.6	7.9	10.4	21.4	22.0	4.4	>4:48.2%
Majidi et al. (Stroke Bridge Clinic), [17] 2017; USA	2013–2014	R	TIA and mIS	NIHSS ≤ 3	Clsc	22	-	4.5	-	-	-, -	-	-	-	-	-	-	-	-	-	-	-
Montassier et al., [18] 2013; France	2009–2009	P	TIA	NA	T. B.	62	1.7	1.7	1.7	1.7	73.1, 46.7	45.0	11.7	-	8.3	21.7	8.3	-	6.7	8.3	1.7	>4:28.3%
Olivot et al. (TWO ACES), [8] 2011; USA	2007–2009	P	TIA and mIS	≥24 h	Clsc	157	-	0.6	0.6	0.6	67, 55.0	49.0	11.0	3.0	-	51.0	16.0	0.7	6.0	-	15.0	Median: 3 [3,4]
Sanders et al. (M3T), [4] 2012; Australia	2004–2007	P	TIA	NA	Clsc	301	-	-	-	2.4	67.7, 58.1	67.4	26.6	-	-	59.5	14.6	14.3	-	-	-	-
Vora et al. (TIA-TEAM), [19] 2015; USA	2010–2011	P	TIA	NA	Clsc	58	0.0	1.7	1.7	1.7	-, -	-	-	-	-	-	-	-	-	-	-	Median: 3 [2–4]
Wasserman et al. (Ottawa), [20] 2010; Canada	2007–2009	P	TIA	NA	Clsc	982	1.0	1.9	2.6	3.2	67, 50.9	58.1	19.9	-	16.6	32.5	8.6	45.0	13.3	-	11.7	>4:67%
Weitzel-Mudersbach et al., [21] 2011; Denmark	2007–2008	P	TIA	NA	Clsc	107	0.9	-	-	-	-, -	-	-	-	-	-	-	-	-	-	-	-

I, inpatient (Medical-Surgical Units); P, prospective; R, retrospective; TIA, transient ischemic attack; mIS, minor ischemic stroke; Clsc, classic definition of TIA; T. B., tissue-based definition of TIA; NA, not applicable; NIHSS, National Institutes of Health Stroke Scale; ABCD2, a clinical score including age, blood pressure, clinical features, duration of TIA, and presence of diabetes.

**Table 2:** Details of the outpatient TIA care models.

Study and Outcome	Patient Stratification Strategy	Management	Clinic Details	Considerations
Chang et al. (RAVEN), [7] 2019; USA Outcome: RAVEN feasibility and safety; 90-day readmission or return to the ED; and risk of TIA, stroke, or death.	**Clinic:** ○ NIHSS < 5, No disabling symptoms based on AHA/ASA criteria. **AND** ○ Absence of RAVEN criteria: hemorrhage in head CT, IV tPA in ED, fluctuating symptoms within the past month, new-onset AFib or cardiac ischemia in ECG, persistent hypertension, use of IV hypertensive agents in ED, large artery stenosis > 50%, and inability to FU within 24 h.	**ED**: ○ Head CT and neurology consult for TIA diagnosis; ○ Vascular neurologist confirmation for ED disposition; ○ ASA 81 mg, HgA1c, and lipid profile. **Clinic**: ○ All patients: carotid duplex and transcranial Doppler. ○ If indicated, MRI, cardiac echocardiogram, and Holter monitoring.	○ Business hours; ○ Vascular neurologist; ○ Automated registry; ○ Patient instruction before discharge; ○ 90-day telephone follow-up (PCP or next of kin contacted if not available); ○ Interval chart reviews.	○ 95.1% of those referred were seen in the clinic < 24 h. ○ 19.1% had a 90-day admission or returned to an ED. ○ 4.3% dual antiplatelet and statin, ○ 38.2% single antiplatelet. ○ 13.5% CTA. ○ 1.2% MRI or MRA. ○ Mimics (44%): 15% peripheral neuropathy, 13% migraine, 4% seizure or transient worsening of previous stroke symptoms, and 2% established final diagnosis.
Cheong et al., [13] 2019; Australia Outcome: 90-day stroke risk.	**Stroke Unit:** ○ >50% stenosis: dual antiplatelet therapy and carotid endarterectomy. ○ No significant stenosis but AFib with or without sub-adequate anticoagulation: initiate anticoagulation. **Clinic:** ○ <50% stenosis on intra- and extra-cranial CTA, low-risk cardioembolic source.	**ED:** ○ Routine lab test, ECG, head CT, and aortic arc to cranial vertex CTA. ○ Initiate ASA (300 mg) or change to clopidogrel (300–600 mg). **Clinic:** ○ MRI (all, within 3–4 days); ○ If CTA is contraindicated, add an additional aortic arch to the Circle of Willis MRI; ○ Medical and lifestyle modification.	○ Business hours; ○ A nurse and stroke registrar, supervised by a vascular neurologist; ○ 90-day telephone FU and medical record search.	○ Only 52% of the admitted patients met the predefined criteria. ○ 250 patients were not referred to the clinic; 47% missed being referred by medical team. ○ Ischemic diagnoses (35%): 11.2% minor stroke, 12% transient symptoms with infarction, 11.6% TIA, 1% persistence symptoms > 24 h, and image negative. ○ Mimics (65%): 16.1% migraine with aura, 11.1% migraine without aura, 8% non-localizable symptoms, 5% seizures, etc.
Correia et al., [14] 2015; PortugalOutcome: 30- and 90-day risk of stroke, TIA, MI, or vascular death among TIA versus possible TIA patients.	○ Not defined.	**ED:** ○ CT, ECG, and blood tests. **Clinic:** ○ Transthoracic/transesophageal echocardiogram, 24-hour Holter monitoring, neck ultrasound, and transcranial Doppler (if indicated).	○ Once a week; ○ Stroke neurologist; ○ FU at 30-day and 90-day; if missed: telephone, hospital, and ED clinical file review; or reach out to PC after 90-day.	○ Excluded patients known to be unable to be followed. ○ Event to clinic visit for TIA: 4.5 days and for possible TIA: 4 days. ○ Mimics (19.7%): predefined criteria for screening; 25.5% presyncope or syncope; 21.1% psychiatric; 17.7% seizure; and other mimics.
Dutta et al., [15] 2016; UK Outcome: 90-day, 1-year, 2-year, and end-of-follow-up risk of stroke, MI, any vascular event (TIA, stroke, or MI), and all-cause death.	○ Not defined.	**Clinic:** ○ All patients: same-day CT scan, carotid duplex ultrasound electrocardiogram, and blood tests. ○ If indicated, MRI, echocardiograms, Holter monitoring, CT, or MR angiograms. ○ All treatment (antiplatelets, statins, antihypertensives, and oral anticoagulants in AFib) was prescribed after the consultation.	○ Business hours. ○ Stroke physician with >7–10 years of stroke experience. ○ Referral from the ED, GP, paramedics, and other departments, such as ophthalmologists. ○ Electronic FU through hospital databases, county registration officers, and funeral directors for capturing deaths without patient contact.	○ 1.3% did not attend the clinic. ○ 14.8% carotid stenosis > 50%, ○ Event to clinic visit of all patients: 4.5 [2–9.3] days, ○ Mimics (50.4%): 69.3% nonspecific transient symptoms with no firm diagnosis; 25.8% migraine; 3.8% transient global amnesia; and 1.8% brain tumors. ○ 34.9-month FU changed the diagnosis in 12 patients.
Harrison et al. (Fast-Track TIA), [10] 2010; UK Outcome: Evaluate the prognostic value of ABCD and ABCD^2^ in subsequent stroke prediction up to 14 years.	○ Database search for identifying TIA and minor stroke patients.	Details are not available.	○ Twice weekly; ○ A consultant stroke physician with >10-years of experience.	○ 15-day median interval from onset to clinic visit. ○ 17.3% stroke risk within 13.8 years FU. ○ ABCD and ABCD2 scores of ≥3 may be useful in identifying TIA outpatients at risk of stroke in the medium to long term.
Lavallée et al. (SOS-TIA), [16] 2017; France Outcome: 90-day and 1-year risk of stroke, MI, or vascular death; compare investigational findings between typical and atypical transient symptoms.	**Clinic:** ○ A trained nurse or senior vascular neurologist screened the patients through a telephone interview with referring health care practitioners.	**Clinic:** Based on a vascular neurologist’s opinion: ○ Brain MRI or CT, extracranial and intracranial arterial evaluation (cervical duplex ultrasonography and transcranial Doppler, plus MRA or CTA), cardiac investigations (ECG and echocardiography), and standard blood chemistry. ○ If visual symptoms are present: ophthalmology evaluation.	○ Outpatient TIA clinic located in a stroke unit. ○ 24/7 clinic accessible through a toll-free phone number. ○ Senior vascular neurologist, one-to-one interview based on a questionnaire. ○ Referral from PCP, cardiologists, ophthalmologists, neurologists, and ED. ○ 90-day telephone or clinic visit (if not available, a close relative or family practitioner was contacted).	○ Event to clinic visit: 1 [0–8] day. ○ 53% were seen in clinic within 24 h of symptom onset. ○ Mimics (29.2%): 33.2% migraine, 23.9% bizarre symptoms, 12.1% unspecified, 7.7% epilepsy, etc.
Majidi et al. (stroke bridge clinic), 2017; [17] USA Outcome: 7-day clinically detectable stroke, TIA, death, and clinical deterioration in the minor stroke subset.	○ RCT for inpatient vs. outpatient care. ○ **Inclusion:** TIA or minor stroke with NIHSS < 3 within 6 h of onset, posterior circulation involvement independent of imaging finding. ○ **Exclusion:** anterior circulation and symptomatic extracranial internal carotid artery stenosis; history or active AFib; and acute medical condition requiring hospitalization.	**ED:** ○ All evaluations are performed by a vascular neurologist. ○ All < 24 h: blood tests, CT, ECG, cardiac telemetry while in the ED. ○ All: statin, antiplatelet (<12 h). ○ Symptoms referable to ant circulation: carotid duplex exam, MRA, and CTA. ○ TTE for all.	○ Vascular neurologist; ○ To be seen in clinic < 72 h of ED discharge; ○ FU at 7–10 days.	○ 81% had MRI; 32% of TIA patients and 95% of stroke patients with MRI had positive DWI. ○ Ant circulation: 100% carotid duplex, 64% CTA, 36% MRA; 9% of TIA and 31% of stroke had anatomically significant disease involving the intracranial large vessels. ○ 91% transthoracic echocardiography. ○ 22 patients (14 minor strokes and 8 TIAs) eligible but decline to participate in the RCT.
Montassier et al., [18] 2013; France Outcome: 90-day TIA, stroke, death, and risk of stroke using ABCD^2^, symptomatic carotid stenosis > 70%, hospitalization for cardiovascular events.	**Clinic:** ○ Recovery of symptoms, unremarkable physical exams, blood tests, head CT, and ECG negative for AFib.	**ED:** ○ Initial diagnosis by ED physician without Neurologist; all underwent blood tests, ECG, and non-contrast CT and received KARDEGIC^®^ 160 mg per day prior to discharge. **Clinic:** ○ Extracranial Doppler testing of supra-aortic arteries; vascular neurologist consultation within 8–15 days.	○ Details are not available; ○ Vascular neurology; ○ 90-day telephone FU and medical record review.	○ 52.5% of TIA patients were discharged from the ED with a referral, and 96.6% of them were managed in the TIA clinic ○ ED discharge to vascular neurology consultation: mean 14.2 days. ○ 100% head CT at the ED. ○ 1% severe carotid stenosis. ○ Doppler mean: 8.1 days after ED discharge.
Olivot et al. (TWO ACES), [8] 2011; USA Outcome: 7-, 30-, and 90-day stroke, MI, and vascular death; program evaluation.	○ ABCD2 0–3: discharge from ED to clinic. ○ ABCD2 4–5: cervical and intracranial imaging in ED; if >50% stenosis: admission; otherwise discharge to clinic. ○ ABCD2 > 5: hospitalization.	**ED:** ○ Non-contrast CT, routine lab studies, and ECG. **Clinic:** ○ MRI and MRA (cervical and intracranial, if contraindicated: head CT, CTA, and carotid US) within 1–2 business days of ED discharge.	○ ED physicians had multiple educational courses and vascular neurologist consulting (telephone). ○ Option for deviation from protocol based on clinical judgment. ○ FU telephone, or routine visit.	○ 70% of patients discharged to TIA clinic; 92% of them were seen <24 h of symptoms’ onset. ○ ED discharge to clinic visit: 3 [2–5] days. ○ 76% MRI before clinic; delay from symptoms’ onset: 3 [2–4] days. ○ 92% vascular imaging before clinic. ○ Mimics (48%): 42% no definite alternate diagnosis, 30% migraine, 8% toxic/metabolic, 6% benign paroxysmal positional vertigo 4% seizure, 3% transient global amnesia, 3% recurrence of previous stroke symptoms, 2% syncope, 1% tumor, and 1% peripheral neuropathy.
Sanders et al. (M3T), [4] 2012; Australia Outcome: 90-day stroke.	○ Rapid management in the ED followed by outpatient care. ○ Before and after study design based on vascular mechanisms and independent of ABCD2. **Stroke unit:** ○ Persistent signs, recurrent/ crescendo TIA, or other acute medical issues. **Clinic:** ○ None of the above symptoms. ○ Priority of referral for ipsilateral internal carotid artery stenosis ≥50% and AFib. ○ Without symptomatic carotid stenosis or AFib: appointment in 4–6 weeks.	**ED:** ○ ED physician evaluation and consultation with the stroke team. ○ All: CT, ECG, and blood tests ○ Same or next day carotid US. ○ Antiplatelets start in ED. **Clinic:** ○ If AFib: anticoagulation assessment. ○ If ≥50% ipsilateral internal carotid artery stenosis: confirmatory CTA or contrast-enhanced MRA within 24 h. ○ If symptomatic stenosis ≥70%: immediate referral for surgical intervention.	○ Neurologist; ○ ED physician fax the oncoming TIA patient to the clinic.	○ Carotid ultrasound (85.5%); ○ 95.9% 90-day FU; ○ Mimics (38.3%).
Vora et al. (TIA-TEAM), [19] 2015; USA Outcome: 7-, 90-, and 365-day risk of TIA, stroke, MI, or death, and compare with DWI and ABCD2 scores for stroke prediction rates.	**Inpatient:** ○ A definite or possible cerebrovascular event (Positive DWI, symptomatic vessel stenosis > 50%, or clinical judgment). **Clinic:** ○ Per on-call neurology resident, confirmed by stroke neurologist.	**ED:** ○ Cardiac and neurologic monitoring up to 23 h in the ED. ○ Multimodal MRI (DWI, FLAIR, GRE, ASL, PWI, and MRA head/neck). ○ If gadolinium is contraindicated: DWI, ASL, and TOF MRA. ○ If MRI is contraindicated: head CT, CTA, and CTP.	○ Outpatient clinics with a trained PCP, general neurologist, or vascular neurologist. ○ Telephone interview for FU; if not responsive, a letter was sent to patients.	○ The median duration from symptom onset to ED arrival was 3.8 [1.6–5.6] hours. ○ 87% underwent acute MRI, and 5% had outpatient MRI after ED discharge. ○ Median delay from onset to acute MRI:16 [10–23] hours. ○ 23% Pos DWI, 8% symptomatic stenosis, and 4% both. ○ Mimics (24%).
Wasserman et al. (Ottawa), [20] 2010; Canada Outcome: 2-, 7-, 30-, and 90-day risk of stroke, TIA, MI, and death.	**Inpatient:** ○ Diagnosis of stroke or TIA mimics and low level of consciousness. **Clinic:** ○ Final diagnosis of TIA in ED, <7 d of onset. ○ Based on ABCD2; ○ High risk (ABCD2 ≥6): <7 d; ○ Moderate risk (ABCD2 =4 to 5: 7–14 d; ○ Low risk (ABCD2 <4) > 14 d.	**ED:** ○ Blood tests, head CT, and ECG. ○ Consult a neurologist if needed. ○ Medication by an ED physician per guidelines from the Stroke Clinic. **Prior to the Stroke Clinic, regardless of risk:** ○ FBS, lipid profile, carotid doppler, echocardiogram, and 24-hour Holter monitoring. **Clinic:** ○ All strata have the same standard of care.	○ Comprehensive Stroke Clinic; ○ Stroke neurologist; ○ Only referred from ED; ○ 90-day FU by valid telephone questionnaire and chart review, neurologist confirmation for stroke occurrence, and 3-physician consult for other outcomes.	○ 92% of TIAs were referred to the Stroke Clinic. ○ Event to clinic: 67.2% < 24 h, 18.7% < 48 h, and 13.4% < 1 week, ○ <1% carotid endarterectomy, ○ 71.5% carotid Doppler, 57.1% Echocardiogram, and 20.2% Holter monitoring within 2 weeks, ○ Excluded from the study: stroke (neurological deficit > 24 h), GCS < 15, TIA mimic, presenting to ED > 7 days following the index event.
Weitzel-Mudersbach et al., [21] 2011; Denmark Outcome: 1-year combined risk of stroke, MI, or vascular death; 1-year secondary prevention compliance; 7- and 90-day and 1-year cumulated stroke risk.	**Inside the primary coverage area:** ○ Crescendo TIA or TIA within the last 48 h: admit in stroke unit for 1–2 days. ○ Otherwise seen in clinics 2–3 within days. **Outside the primary coverage area:** ○ Refer to clinic after acute admission on a local medical ward or referral from a GP.	**Stroke Unit:** First outpatient visits or 24 h in the stroke unit: ○ Brain CT/MRI, ECG, blood tests, Duplex sonography of the extra- and intracranial vessels, and ankle/brachial index. ○ For all 150 mg aspirin (150 mg). ○ All TIA: long-term aspirin 75 mg and dipyramidole 200 mg twice daily starting at day 2. ○ If not tolerated aspirin, dipyramidole, or PAD: clopidogrel 75 mg/day. ○ If AFib: warfarin. ○ If AFib and symptomatic arterial stenosis: warfarin and 300 mg clopidogrel, or carotid endarterectomy within 1 week. ○ AFib without or inadequate coagulation: heparin with low molecular weight. ○ When indicated: cholesterol lowering, anti-hypertensive treatment, and lifestyle modification.	○ Referred to the clinic by a GP, neurologists, ophthalmologists, or EMS, bypassing the ED. ○ Phone interview by a trained nurse at 7 and 90 days, and a clinical visit by a stroke neurologist after 1 year.	○ Inclusion of TIA with mRS ≤ 2; ○ 35% were seen as outpatients; ○ From index TIA, 58.5% were seen within 24 h and 76.1% within the first week; ○ 8.1% carotid endarterectomy in all study cohorts.

ED, emergency department; TIA, Transient ischemic attack; MI, myocardial infarction; NIHSS, NIH Stroke Scale score; mRS, modified Rankin Score; GCS, Glasgow coma scale; Afib, atrial fibrillation; tPA, tissue plasminogen activator; GP, general practitioner; PCP, primary care physician; FU, follow up; PAD, Peripheral Arterial Disease; MRI, magnetic resonance imaging; MRA, magnetic resonance angiography; CTA, computed tomography angiography; ECG, electrocardiogram; ASA, acetylsalicylic acid; TTE, transthoracic echocardiogram; DWI, diffusion weighted imaging, fluid attenuated inversion recovery; gradient echo sequences; ASL, arterial spin labeling; perfusion weighted imaging; TOF, tetralogy of Fallot; CTP, computed topography perfusion.

## Data Availability

Not applicable.
